# Exploring the relation between mortality and left ventricular structure and function in stable hemodialysis treated patients, a longitudinal multicenter cohort study

**DOI:** 10.1038/s41598-021-91431-9

**Published:** 2021-06-16

**Authors:** Lazar A. Chisavu, Adrian Apostol, Gheorghe N. Pop, Viviana Ivan, Oana Schiller, Flaviu Bob, Luciana Marc, Adelina Mihaescu, Florica Gadalean, Iulia Grosu, Bogdan Timar, Adalbert Schiller

**Affiliations:** 1 Department of Internal Medicine II – Division of Nephrology, “Victor Babes” University of Medicine and Pharmacy Timisoara, Timisoara, Romania; 2 Centre for Molecular Research in Nephrology and Vascular Disease, Faculty of Medicine, “Victor Babes” University of Medicine and Pharmacy, Timisoara, Romania; 3 Avitum BBraun, Timisoara, Romania; 4 Department of Internal Medicine II – Division of Cardiology, “Victor Babes” University of Medicine and Pharmacy Timisoara, Timisoara, Romania; 5 Cardiology Clinic- County Emergency Hospital “Pius Brinzeu”, Timisoara, Romania; 6 Department of Cardiology VI – Division of Outpatient Internal Medicine, Cardiovascular Prevention and Recovery, “Victor Babes” University of Medicine and Pharmacy Timisoara, Timisoara, Romania; 7 Department of Functional Sciences, Centre for Modeling Biological Systems and Data Analysis, “Victor Babes” University of Medicine and Pharmacy, Timisoara, Romania; 8 Nephrology Clinic - County Emergency Hospital “Pius Brinzeu”, Timisoara, Romania; 9 Diabetes and Metabolic Diseases Clinic - County Emergency Hospital “Pius Brinzeu”, Timisoara, Romania

**Keywords:** Cardiology, Nephrology

## Abstract

Left ventricular (LV) structure and function anomalies are frequent during the CKD continuum and are associated with increased risk of mortality. Cross section and longitudinal ultrasound data are available for advanced CKD and transition to ESKD. Less information is available about LV changes during stable, long-term hemodialysis (HD) treatment. All stable HD patients from 9 HD centers (1034 patients, 671 males, age 58.71 ± 12.94 years) have been enrolled in January 2015. The cohort was followed-up for 4 years, kidney transplantation or death. Yearly, two-dimensional and M-mode continuous and Pulse Doppler echocardiography were performed. During the follow-up, the prevalence of cardiovascular comorbidities significantly increased (p < 0.0001), coronary artery disease (CAD) from 73.5 to 88.8%, peripheral artery disease (PAD) from 29 to 40.9%, cerebral vascular disease (CVD) from 20.4 to 30.8%, heart valves calcification (VC) from 65.6 to 89.3% and left ventricular hypertrophy (LVH) from 67.6 to 76.5%. The mortality risk increased with the presence of CAD (1.59-fold), PAD (1.61-fold), CVD (1.59-fold), and VC (1.77-fold). Mortality risk was increased in those with LVEF < 50% (LVEF 40–49% 1.5-fold and LVEF < 40% 2.3 fold). Among the survivors of the first year, LVEF varied (> 5% decrease, > 5% increase and ± 5% variations). More than 5% increase of LVEF was associated with higher mortality risk (crude 1.5-fold, adjusted 1.43-fold) compared to stationary EF (p = 0.001). Cardiovascular disease progresses during stable long-term HD therapy and increases mortality risk. HF becomes highly prevalent but only HF with decreased LVEF < 50% is associated with increased risk of mortality.

## Introduction

The prevalence of cardiovascular disease (CVD) in chronic kidney disease (CKD) patients is twofold higher as compared to the no CKD general population (65.1% vs. 32.6%) and it increases with age and kidney function loss. Therefore, in end stage renal disease (ESRD), cardiovascular comorbidities are common (overall prevalence 70.6%) even in young patients^1^.

The mortality of hemodialysis (HD) treated ESRD patients is very high compared to the general population (in Europe 8.8-fold higher for CVD and 8.1-fold higher for non-cardiovascular causes)^2^. In these patients, CVD remains the main cause of death. The renal registries (The USRDS, UK RR) and cohort studies (2014 Swedish cohort) reported a CVD related mortality varying between 27 and 69%^1,3,4^.

In advanced CKD and ESRD, left ventricular structure and function anomalies (left ventricular hypertrophy, low ejection fraction and so on) investigated by a large variety of imaging methods, proved to be associated with high cardiovascular and all-cause mortality risk^5–7^. Most of the available data are based on cross-sectional design studies or post hoc analysis of trial participants. The longitudinal study of a subset of CRIC participants exploring left ventricular structure and function at transition from advanced CKD to HD (2013)^8^ and the Japanese observational cohort study from 2013, exploring longitudinal evolution of vascular and heart valve calcifications in HD patients^9^ are among the few exceptions.

Less is known about long-term HD therapy and its effects on myocardial structure and function under the novel conditions: anemia and erythropoietin stimulating agents (ESA) therapy, chronic kidney disease–mineral bone disorder (CKD-MBD) and its treatment, fluid overload, novel blood pressure conditions, novel diet and metabolism conditions, and so on.

The aim of our multicenter longitudinal observational cohort study was to continue to explore left ventricular function and structure changes and their impact on mortality in stable HD treated ESRD patients. Also, in this study, we highlight clinical and laboratory predictors of adverse events. We describe mortality according to baseline hearth failure and also describe the impact of the changes in the value of EF on mortality.

## Methods

All stable HD treated ESRD patients (> 90 days HD therapy) from 9 HD centers from Romania have been enrolled in the study in January 2015. The cohort consisted of 1034 patients, (671 male) average age 58.71 ± 12.94 years. Personal data, history of disease, HD therapy parameters were retrieved from patient’s dialysis files. Diabetes mellitus (DM) was evidenced in 20.9% patients at inclusion. HD therapy was performed using high flux, high surface dialyzers, 3 times/week (≥ 12 h/week) in all cases. The assessment, therapy and follow-up of anemia and CKD-MBD were performed according to KDIGO guidelines. The cohort was followed-up for 4 years or kidney transplantation or death. The study was approved by the Ethics Committee, of the Avitum BBraun Romania Dialysis Center, and all patients signed a written informed consent. The study was in accordance with the Ethics Code of the World Medical Association.

### Cardiology assessment

Yearly, two-dimensional and M-mode continuous and Pulse Doppler echocardiography were performed in accordance with the recommendations of the European Association of Cardiovascular Imaging (EACI), between the 2nd and 3rd hour of dialysis session, by the same operator using the same device (in order to avoid inter-observer differences). After a regular exam of cardiac morphology and function, we assessed the left ventricular ejection fraction (LVEF) by using the Simpson method, and we noted the presence of heart valve calcifications. Heart failure (HF) was assessed by the presence of clinical features (breathlessness, fatigue, ankle edema) and with structural and/or functional anomalies of the heart according to the European Guidelines on Acute and Chronic Heart Failure^10^.

According to these guidelines, patients were divided into 4 groups:

1. No HF (NHF): no clinical features, EF not altered.
2. HF with preserved EF (HFpEF): presence of symptoms/signs and LVEF ≥ 50%.
3. HF with mid-range EF (HFmrEF): presence of symptoms/signs and LVEF 40–49%.
4. HF with reduced EF (HFrEF): presence of symptoms/signs and LVEF < 40%.

Diagnosis of coronary artery diseases (CAD), Cerebrovascular Disease (CVD) and peripheral artery diseases (PAD) was assessed according to the guidelines^11–13^.

### Statistical analysis

Data are presented as average ± standard deviation (numerical variables with Gaussian distribution), median and interquartile range (numerical variables with non-Gaussian distributions) respectively percentage from the sub-group total and number of individuals. Continuous variables distributions were tested for normality using Shapiro–Wilk test, and for equality of variances using Levene’s test. We employed ANOVA test in order to check if age introduced a survival bias. The individual impact of several confounding factors on the variance of a continuous variable was assessed by building multivariate regression models. The quality of the model was described using the accuracy of prediction and by Nagelkerke’s R2. The predictors, in the final regression equations, were accepted according to a repeated backward-stepwise algorithm (inclusion criteria p < 0.05, exclusion criteria p > 0.10) in order to obtain the most appropriate theoretical model to fit the collected data. For assessing survival, we employed Kaplan–Meier survival curves with the Breslow test for pairwise comparison. We only right-censored at the time of kidney transplant or 1 January 2019. The end-point of this study was death. We test our data for proportional hazard assumption and since it was not violated, we continue with Cox proportional hazard regression models that were employed to estimate hazard ratio.

In this study, a p-value of 0.05 was considered the threshold for statistical significance. Data were analyzed using SPSS v26 statistical software package (SPSS Inc, Chicago, IL, USA) for Linux.

## Results

The baseline characteristics of the investigated cohort are presented in Table 1.

**Table 1:** General characteristics of the cohort at inclusion (n = 1034).

Age (years)	58.71 ± 12.94
Male gender	671 (60.8%)
Average HD vintage (years)	3.56 [1.8–5.96]
Dry weight (av) (kg)	70.5 [60–83.45]
eKt/V (av)	1.46 [1.27–1.62]
EF (av) (%)	57.69 ± 9.54
Hb (av) (g/dL)	11.1 [10.4–11.9]
Ferritin (av) (ng/mL)	729 [508.6–1006.3]
TSAT (av) (%)	31 [23–44.4]
CRP (av) (mg/L)	1.86 [0.58–6.41]
Albumin (av) (g/dL)	4 [3.73–4.21]
Ca (av) (mg/dL)	8.74 [8.3–9.2]
PO4 (av) (mg/dL)	4.7 [3.74–5.7]
iPTH (av) (pg/mL)	314.1[143.2–600.7]
HCO3- (av) mmol/L	21.2 [19.2–23.12]
HD performed on catheter (n + %)	191 (18.4%)

*av* average, *kg* kilograms, *eKt/V* effective Kt/V, *EF* ejection fraction, *Hb* Hemoglobin, *g/dL* grams/deciliter, *ng/mL* nanograms/milliliter, *TSAT* transferrin saturation, *CRP* C-reactive protein, *Ca* calcium, *mg/dL* miligrams/deciliter, *PO4* phosphorus, *iPTH* intact parathyroid hormone, *pg/mL* picograms/militer, *mmol/L* milimols/liter, *HD* hemodialysis.

The prevalence of the main comorbidities has been assessed yearly. At the end of the study, all cardiovascular comorbidities were significantly more prevalent compared to inclusion (p < 0.0001): CAD—88.8% vs 73.5%, PAD—40.9% vs 29%, CVD—30.8% vs 20.4%, Vascular/heart valve calcification (VC) 89.3% vs 65.6%, Left ventricular hypertrophy (LVH) 76.5 vs 67.6%. During the 4-year follow-up time, all-cause mortality was 25.3% (261 patients died). The survival rate in the first year was 90.5% (98 deaths), in the second it was 85.9% (48 deaths), in the third it was 77.3% (59 deaths) and in the fourth 74.7% (56 deaths).

In order to assess the independent factors that predict the risk of death in our cohort, we employed a backward multivariate logistic regression model. In our models we included age, gender, dialysis parameters (vintage, dry weight, eKt/V), along with the laboratory and the echocardiographic results. Akaike information criteria (AIC) was used in order to determine the best model. Odds ratio and 95% confidence interval were calculated. Our regression equation proved to be a good fit for the model, explaining 28.6% of death event (R^2^ = 0.286). The risk of death increases with the presence of CAD (by 1.59-fold), of PAD (by 1.61-fold), of CVD (by 1.59-fold) and VC (by 1.77-fold). Increased eKt/V, higher ejection fraction and dry weight turned out to decrease the risk of mortality (Table 2).

**Table 2:** Independent factors that predict the risk of death (n = 1034).

Variable	B	S.E	p	OR	95% OR	
CAD	0.469	0.224	0.036	1.599	1.031	2.480
PAD	0.479	0.196	0.015	1.614	1.098	2.372
CVD	0.468	0.207	0.024	1.597	1.064	2.397
VC	0.576	0.181	0.001	1.778	1.248	2.535
eKt/V	− 0.761	0.318	0.017	0.467	0.251	0.870
EF	− 0.017	0.008	0.024	0.983	0.969	0.998
Dry weight	− 0.019	0.005	0.001	0.981	0.971	0.992

*CAD* coronary artery disease, *PAD* peripheral artery diseases, *CVD* cerebrovascular diseases, *VC* valve calcifications, *eKt/V* effective Kt/V, *EF* ejection fraction.

Before employing survival analysis, we check for survival bias. We split our participant into age groups, the bin size was 10 years of age (18–27, 28–37...78–87). ANOVA test shown no statistically difference of survival between age groups (p = 0.204). Also, the female group in this study was not under represented, similar incidence of male/female ratio is present in European population^14^.

**Figure 1:**
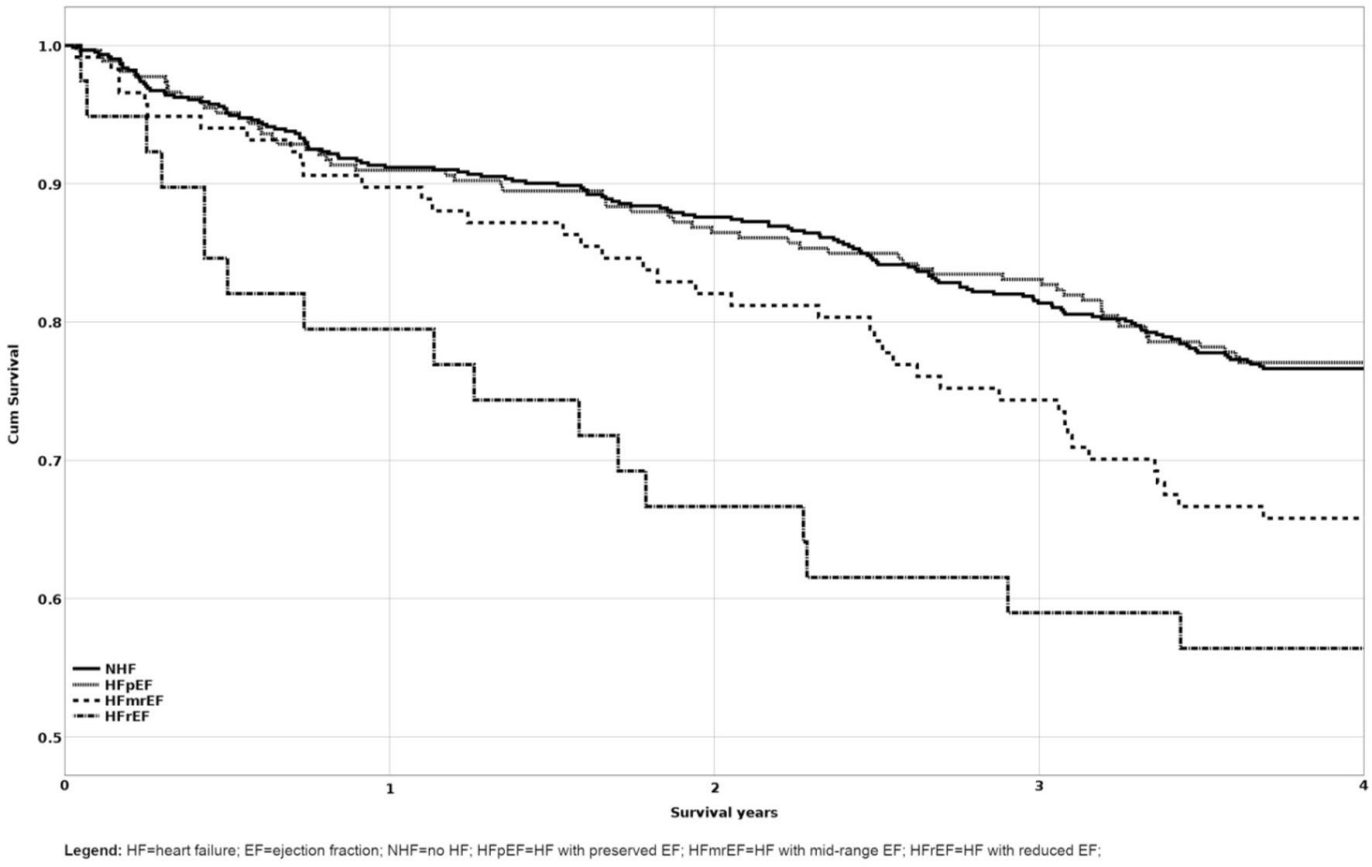
Kaplan Meier survival curves of EF groups for all-cause mortality.

The patients from our cohort were assigned to the four HF groups as described in methods: NHF (n = 612), HFpEF (n = 266), HFmrEF (n = 118) and HFrEF (n = 38). Kaplan-Meier survival analysis was conducted to compare the four groups (Fig. 1). A similar percentage of censored cases was present in the group NHF (76.6%) and HFpEF (77.1%), while in the HFmrEF and HFrEF groups was 66.1% and 55.3% respectively. Patients in the NHF and HFpEF groups had a similar estimated survival, 1270 days and 1269 days respectively, while in the HFmrEF and HFrEF groups estimated survival was lower, 1194 days and 1009 days.

A Breslow test was conducted to determine if there were differences in the survival distribution in the four groups. The survival distribution for the four groups were statistically different, (χ^2^(3) = 17.963, p < 0.001). Pairwise comparisons were conducted to determine which group had different survival distribution and the results are presented in Table 3.

**Table 3:** Breslow pairwise comparisons of survival distributions by HF groups (n = 1034).

Groups	NHF		HFpEF		HFmrEF		HFrEF	
	χ^2^	p	χ^2^	p	χ^2^	p	χ^2^	p
NHF	–	–	0.010	0.919	5.569	0.018*	13.109	< 0.001***
HFpEF	0.010	0.919	–	–	4.676	0.031*	11.532	< 0.001***
HFmrEF	5.569	0.018*	4.676	0.031*	–	–	2.897	0.089
HFrEF	13.109	< 0.001***	11.532	< 0.001***	2.897	0.089	–	–

*HF* heart failure, *EF* ejection fraction, *NHF* no HF, *HFpEF* HF with preserved EF, *HFmrEF* HF with mid-range EF, *HFrEF* HF with reduced EF.

*; **; ***Statistically significant differences (p < 0.05; p < 0.01; p < 0.001).

The mortality risk was analyzed using multivariate Cox proportional hazards models (Table 4). There was no significant difference between patients without HF and those with HFpEF. Lower EF was associated with increased risk for death, HFmrEF group had a 1.5-fold risk while the HFrEF had 2.3-fold risk for death compared to those without HF.

**Table 4:** Cox proportional hazard model of HF groups (n = 1034).

Group	B	S.E	p	OR	95% OR	
HFpEF	− 0.019	0.153	0.903	0.981	0.702	1.324
HFmrEF	0.429	0.179	0.016	1.536	1.082	2.181
HFrEF	0.865	0.257	0.001	2.375	1.436	3.927

*Group* NHF used as reference.

*HF* heart failure, *EF* ejection fraction, *NHF* no HF, *HFpEF* HF with preserved EF, *HFmrEF* HF with mid-range EF, *HFrEF* HF with reduced EF.

**Figure 2:**
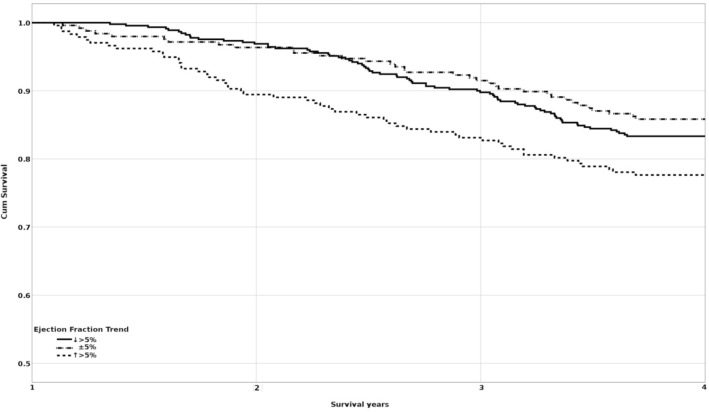
Kaplan Meier survival curves of EF trend groups for all-cause mortality.

During the follow-up time, we evidenced variations of the EF. In order to assess the significance of these variations, EF trends were calculated by the difference between initial EF and EF values after the first year of follow-up. According to the results, we divided the cohort into three groups. In the first group the EF varied between − 5 and + 5% from the initial values (n = 424), in the second EF decreased more than 5% (n = 262), and the third EF increased more than 5% (n = 250). EF trend analysis included patients who survived at least one year in order to get a second ultrasound investigation (n = 936). We observed that the group where EF increased more than 5% had a higher number of events, with a 76.4% survival rate compared with the ± 5% group (85.8%) and the > 5% EF decrease group (83.2%). (Fig. 2).

A Breslow test was conducted in order to determine if there were differences in the survival distribution in the three groups. The survival distribution for the three groups was statistically different, χ^2^ = 11.592; p = 0.003. Pairwise comparisons were performed to determine which group had different survival distribution and the results are presented in Table 5.

**Table 5:** Breslow pairwise comparisons of survival distributions by EF trend (n = 936).

Groups	↓ > 5%		± 5%		↑ > 5%	
	χ^2^	p	χ^2^	p	χ^2^	p
↓ > 5%	–	–	0.741	0.389	4.760	0.029*
± 5%	0.741	0.389	–	–	10.984	0.001**
↑ > 5%	4.760	0.029*	10.984	0.001**	–	–

*; **; ***Statistically significant differences (p < 0.05; p < 0.01; p < 0.001).

The mortality risk for the three groups was analyzed using multivariate Cox proportional hazards models (Table 6). As was presented earlier, HF groups have different survival rate (confounding factor), reason for why we adjusted our regression model also by initial EF. A reduction of more than 5% in EF presented a 0.8-fold risk, but without statistical significance. Patients with an increase of more than 5% in EF had a crude 1.5-fold risk, and an adjusted 1.43-fold risk for death compared to those with a stationary EF (p = 0.001).

**Table 6:** Cox proportional hazard model of EF trend (n = 936).

	Number of deaths	Number of deaths/100 person-years	Hazard ratio (95% CI)	
			Unadjusted	Adjusted*
± 5% EF (reference)	59	4.21	1	1
↓ > 5%	46	4.12	0.83 (0.55–1.24)	0.84 (0.56–1.25)
↑ > 5%	58	5.8	1.50 (1.06–2.13)	1.43 (1.01–2.04)

*Adjusted for age, gender and initial EF.

When we stratify the EF trend by the HF sub-groups, we observe that the group ↑ > 5% EF change has a worse survival rate in HFpEF, HFmrEF and HFrEF sub-groups (Table 7).

**Table 7:** EF trend survival rate by HF sub-groups.

HF	EF trend	n	No. of events	Survival rate
NHF	↓ > 5%	243	41	202 (83.1%)
	± 5%	260	40	220 (84.6%)
	↑ > 5%	55	8	47 (85.5%)
HFpEF	↓ > 5%	16	1	15 (93.8%)
	± 5%	126	14	112 (88.9%)
	↑ > 5%	100	22	78 (78%)
HFmrEF	↓ > 5%	3	2	1 (33.3%)
	± 5%	30	5	25 (83.3%)
	↑ > 5%	3	21	52 (71.2%)
HFrEF	↓ > 5%	–	–	–
	± 5%	8	1	7 (87.5%)
	↑ > 5%	22	8	14 (63.6%)

## Discussion

Since 2003 (JNC 7) CKD is considered a major risk factor for CVD. As CKD progresses, the prevalence of conventional CVD risk factors increases and novel (CKD related) risk factors emerge^15^. Therefore, in advanced CKD the prevalence of CVD and CVD related mortality is very high. Cardiac structure and function anomalies are detected in earlier stages of CKD. In stage 3 CKD, traditional echocardiography and 2D strain analysis revealed altered left atrial systolic and late diastolic strain rates, and enlarged indexed left atrial volume. Left ventricular late diastolic strain rate turned out to be reduced suggesting decreased left atrial contractile function^16^. Left ventricular hypertrophy (LVH), increased left ventricular mass index (LVMI), diastolic dysfunction, decreased left ventricular ejection fraction (LVEF) are common in pre dialysis CKD patients and associated with poor cardiovascular outcome^17^. In CKD patients, even with normal LVEF, early detection of impaired LV myocardial function was possible by two and three-dimensional speckle-tracking echocardiography and was associated with higher CV risk^7,18^. The high mortality rate in advanced CKD and ESKD was related to LV dyssynchrony also^19^. Myocardial structure and function anomalies progress along the CKD continuum related to hypertension, fluid overload, anemia, uremic toxins, myocardial fibrosis, coronary artery disease (CAD)and so on^20^.

Initiation of hemodialysis (HD) in ESKD seems to change some of these risk factors by reducing blood pressure, eliminating some of the uremic toxins, better controlling the fluid overload, acid–base and electrolyte balance. At the same time some other risk factors emerge, excess of endotoxins, consequences of the AV fistula, myocardial stunning, HD induced hemodynamic stress^21^. All those changes are influencing myocardial function and structure and may change ultrasound findings also. Indeed, initiation of HD induced decrease of LVMI, concentric or eccentric remodeling of LVH in heart failure HD patients^22^. In preserved LVEF patients, 2D strain analysis showed a better left ventricular function in HD patients as compared to advanced CKD ones^7,23^. Based on these findings, early initiation HD was proposed. The Echo sub study of the IDEAL trial failed to evidence any heart ultrasound traceable benefits in the early initiated patients compared to the late initiated ones (GFR 10–14 vs 5–7 mL/min/1.73 m^2^)^24^. The meta-analysis of 46 small studies concerning the effects of more intensive HD therapy (more frequent, longer HD sessions) showed improvement of myocardial function and morphology in the intensive group. No cardiovascular survival benefits were explored in the study^25^.

The first study following up the ultrasound structure and function changes from advanced CKD to ESRD (HD) was performed on a subset of patients from the CRIC (Chronic Renal Insufficiency Cohort). The ultrasound findings one year prior to HD initiation and one year HD therapy evidenced no significant changes in LVMI but a significant decrease of LVEF^8^.

According to our knowledge this multicenter longitudinal study is the first to explore changes of myocardial function and structure in a large cohort of 1034 stable HD patients (average HD vintage at inclusion 3.56 years). As expected, during the 4 year follow-up the prevalence of clinically evident CAD, PVD and CVD significantly increased (73.5% to 88.8%, 29% to 40.9% and 20.4% to 30.8% respectively—all p < 0.0001). On echocardiography, these data have been associated with increasing prevalence of LVH from 67.6 to 76.5% and of heart valve calcifications from 65.6% to 89.3% (all p < 0.0001). We should emphasize the very high and increasing prevalence of VC in our cohort as compared to the USRDS data (35 to 40%)^26^. As recently suggested, during long term HD therapy, the cumulative effect of many novel cardiovascular risk factors (uremic toxins, oxidative stress, endothelial dysfunction, chronic inflammation, protein carbamylation, anemia, CKD-MBD and so on), play an important role in the progression of cardiovascular disease and of cardiac structure anomalies^27^.

In the four year follow-up time 261 patients died (all cause cumulative mortality was 25.3%). The survival rate decreased, being 90.5%, in the first year, 85.9%, in the second, 77.3% in the third and 74.7% in the fourth. The risk of death increased with the presence of CAD by 1.59-fold, with the presence of PAD by 1.61-fold, of CVD by 1.59-fold and of VC by 1.77-fold. It seems that increased eKt/V and dry weight, higher LVEF were associated with decreased risk of mortality. Similar data have been reported in Japan in patients over 10-year treatment with HD^28^.

The average LVEF at inclusion was 57.69 ± 9.54% and 15.08% of the cases presented a LVEF < 50%. We assigned the patients to the groups presented in methods^10^. The patients in the groups with LVEF > 50% had a significantly higher estimated survival as compared to those with LVEF < 50% (groups 1 and 2, 1270 and 1269 days respectively vs. group 3 and 4, 1194 days and 1009 days). The mortality risk was also higher in the group 3 (1.5-fold) and 4 (2.3-fold). Reduced LVEF at starting of HD represent a strong independent predictor of cardiovascular death^29^. 40.8% of the patients had heart failure criteria according to 2016 ESC guidelines^10^. In contrast to some of the prior publications^30^, estimated survival was lower and risk of death were higher only in the groups with LVEF < 50%, meaning in 15.08% of the entire cohort (excluding the HFpEF group). Our results in a cohort of ESKD patients treated with HD are similar to those published by the MAGGIC group meta-analysis^31^.

At the second echocardiography performed 1 year after inclusion in the cohort, in the first year survivors (n = 936), we detected some variations in the LVEF. In order to assess the significance of those variations, we divided the cohort into 3 groups: a group with > 5% decrease of LVEF (n = 262), a group with less than 5% variation of LVEF (n = 424) and a group with more than 5% increase of the LVEF (n = 250). Using the group with less than 5% variation of LVEF as reference, the Cox regression analysis evidenced an increase of mortality risk 1.43-fold in the group with more than 5% increase of EF, when adjusted for age, gender and initial LVEF. In the group of patients with > 5% decrease of LVEF the mortality risk was 0.84 (not significant). LVEF is a risk stratifier for all cause and cardiovascular mortality in HF patients^32^. One could expect a decrease of risk with the increase of EF. On the contrary, in our cohort, the increase of EF was associated with an increase of mortality risk. We hypothesized that an increase of LVEF was induced by more or less permanent heart valve regurgitation due to fluid overload, changing pressure regimen on both sides of the valves, accelerated progression of CAD, valve calcification induced by CKD-MBD, uremic cardiomyopathy, all common in HD patients. There were no correlations between the EF trend and vascular calcifications. In these cases, maybe global longitudinal strain alteration should be used as risk marker of cardiovascular mortality, as we have already mentioned.

Changes in ejection fraction were noticed as consequences to ischaemic condition—myocardial infarction or ischemia, and to valvular changes and so-called improvement of EF was due to volume overload in augmented regurgitated flow. We tried to assess EF in hemodynamic stable condition, in patients with normal heart rate 60–80 b/min and stable blood pressure.

We want to stress the importance of EF changes during time, EF might vary consistently due to volume overload of cardiac origin in the case of valvulopathies or of dialysis origin in the overhydrated patients. Also, this parameter might vary in arrhythmias or acute conditions, so our evaluation was done in stable condition. An improvement of EF might not be always a marker of good prognosis.

## Conclusions

Cardiovascular disease progresses during stable long-term HD therapy and increases mortality risk. The novel (HD related) cardiovascular disease risk factors seems to further increase prevalence of LVH and decrease LVEF. HF becomes highly prevalent in long term HD treated patients but only HF with decreased LVEF < 50% is associated with increasing risk of mortality. More or less permanent increase in LVEF, under these conditions, being associated with increased mortality risk, may reflect a dynamic process of volume compensated increase of heart valves/contractility dysfunction.

These findings support the idea of repeated echocardiographic evaluation in order to reveal as soon as possible changes in morphology with hemodynamic consequences.

### Weak points

The cardiac evaluation was made during the dialysis sessions. The adherence of HD patients to long term follow-up studies needing repeated investigation effort is low as suggested by others. Therefore, we applied the settings suggested by other authors also. The blood pressure was not evaluated during the dialysis session due to the fact that it is highly variating during the procedure. We didn’t evaluate the valvular changes, but the changes are relatively small on the yearly evaluation.

### Strong points

The number of patients is high and the follow-up period is significant (on average half of the patient’s life expectancy).
